# Assessment of animal African trypanosomiasis (AAT) vulnerability in cattle-owning communities of sub-Saharan Africa

**DOI:** 10.1186/s13071-016-1336-5

**Published:** 2016-01-30

**Authors:** H. R. Holt, R. Selby, C. Mumba, G. B. Napier, J. Guitian

**Affiliations:** London Centre for Neglected Tropical Disease Research, London, UK; Department of Production and Population Health, Royal Veterinary College, Hawkshead Lane, North Mymms, Hatfield, Hertfordshire AL9 7TA UK; Department of Vector Biology, Liverpool School of Tropical Medicine, Pembroke Place, Liverpool, L3 5QA UK; School of Veterinary Medicine, University of Zambia, Great East Road Campus, P.O. Box 32379, Lusaka, Zambia; Global Alliance for Livestock Veterinary Medicines (GALVmed), Doherty Building, Pentlands Science Park, Bush Loan, Edinburgh, EH26 0PZ UK

**Keywords:** Animal African trypanosomiasis, Sub-Saharan Africa, Cattle, Tsetse, Vulnerability assessment

## Abstract

**Background:**

Animal African trypanosomiasis (AAT) is one of the biggest constraints to livestock production and a threat to food security in sub-Saharan Africa. In order to optimise the allocation of resources for AAT control, decision makers need to target geographic areas where control programmes are most likely to be successful and sustainable and select control methods that will maximise the benefits obtained from resources invested.

**Methods:**

The overall approach to classifying cattle-owning communities in terms of AAT vulnerability was based on the selection of key variables collected through field surveys in five sub-Saharan Africa countries followed by a formal Multiple Correspondence Analysis (MCA) to identify factors explaining the variations between areas. To categorise the communities in terms of AAT vulnerability profiles, Hierarchical Cluster Analysis (HCA) was performed.

**Results:**

Three clusters of community vulnerability profiles were identified based on farmers’ beliefs with respect to trypanosomiasis control within the five countries studied. Cluster 1 communities, mainly identified in Cameroon, reported constant AAT burden, had large trypanosensitive (average herd size  = 57) communal grazing cattle herds. Livestock (cattle and small ruminants) were reportedly the primary source of income in the majority of these cattle-owning households (87.0 %). Cluster 2 communities identified mainly in Burkina Faso and Zambia, with some Ethiopian communities had moderate herd sizes (average = 16) and some trypanotolerant breeds (31.7 %) practicing communal grazing. In these communities there were some concerns regarding the development of trypanocide resistance. Crops were the primary income source while communities in this cluster incurred some financial losses due to diminished draft power. The third cluster contained mainly Ugandan and Ethiopian communities which were mixed farmers with smaller herd sizes (average = 8). The costs spent diagnosing and treating AAT were moderate here.

**Conclusions:**

Understanding how cattle-owners are affected by AAT and their efforts to manage the disease is critical to the design of suitable locally-adapted control programmes. It is expected that the results could inform priority setting and the development of tailored recommendations for AAT control strategies.

## Background

Tsetse (*Glossina* spp.) and animal African trypanosomiasis (AAT) are an important constraint to livestock production and a threat to food security in sub-Saharan Africa [1]. The production losses in cattle due to trypanosome infections have been estimated to be up to 20 % across a range of parameters, including mortality, calving rate, draft power, meat and milk production [2]. A high tsetse-trypanosome burden constrains the use of land for livestock production, with farmers in these areas often being more reliant on crop farming. However, trypanosomiasis also compromises crop production by reducing the availability of draft animals to plough fields and provide manure for fertiliser [2].

The impact of AAT can be reduced by trypanocide application and the introduction of trypanotolerant cattle breeds. There is no vaccine available for the disease, and reduction in transmission rates is largely reliant on control of the tsetse vector by methods such as insecticide treatment of cattle (ITC), the use of traps or targets, ground or aerial insecticide spraying, or reducing the risk of exposure through changes in livestock management. The process of privatisation of veterinary services in many sub-Saharan African countries means that farmers and community animal health workers (CAHW) with limited training are often responsible for the treatment of the disease [3]. Traditionally, farmer-based control of AAT has relied heavily on the individual use of chemotherapy and chemoprophylaxis, while methods requiring collective action have often been neglected. Trypanosome species, however are becoming increasingly resistant to these common-place treatments [4, 5].

In recognition of the need for co-ordinated actions against AAT, the Pan-African tsetse and trypanosome eradication campaign (PATTEC), funded by the African Development Bank, was established in the year 2000 and has set tsetse elimination as its goal. Although this goal presents a huge challenge that would require extensive resources and there is debate as to whether it is feasible, the last decade has seen renewed interest in the research and development of control options. Governments, charities and philanthropists have made funding available for this purpose, despite this, the reality is that many of the communities afflicted by AAT have insufficient resources available for its control and are not always reached by control programmes. In addition, macro-level decision making may ignore important heterogeneities between communities.

In order to optimise the allocation of resources for AAT control, decision makers target geographic areas where control programmes are most likely to be technically, economically, socially and environmentally sustainable and select methods of control that will maximise the benefits obtained from resources invested [6]. To this end, there is increasing interest in the development of decision-support tools for AAT control. These can be based on a description and analysis of geographical features by means of geographic information systems [7, 8], economic analysis [9, 10], modelling tsetse population dynamics [11] or a combination of these tools [6, 12]. However, the feasibility of applying such tools at local-level may be compromised by the skill and resource base of potential end-users and data availability. Recently, relatively simple frameworks have been proposed to identify the most appropriate control options for AAT based on a small number of ‘key’ indicators of the eco-epidemiological cycle and the cattle rearing system [13]. Although these are useful tools, the impact that AAT has on a community is the result of complex interactions between environmental, political, socio-cultural, entomological and livestock management factors [13]. As a result, further development of existing decision tools, to reflect not only the biological, environmental or technical applicability of disease control, but also the likely impacts on the communities living within affected areas is warranted so that control programmes reach those who are most vulnerable. This study was therefore carried out to use cattle owner interview data to perform a community-level vulnerability assessment, to add to the growing body of evidence for decision making regarding AAT control by identifying typologies or profiles of the communities in terms of their AAT vulnerability. It is expected that the results could inform priority setting and the development of tailored recommendations for AAT control strategies

## Methods

A series of interviews were conducted with cattle owners in different agro-ecological zones across five countries in sub-Saharan Africa, namely Burkina Faso, Cameroon, Ethiopia, Uganda and Zambia. Data collected on cattle was mainly on owners’ knowledge and perceptions of AAT. The study sites provided a large variation in environment, AAT eco-epidemiology, cattle management and socio-economic impact of AAT. The overall approach to classifying communities in terms of AAT vulnerability was based on the selection of key variables collected through field surveys followed by a formal Multiple Correspondence Analysis (MCA) to identify factors explaining the variation between areas. Hierarchical Cluster Analysis (HCA) was then performed in order to categorise communities into groups describing their AAT vulnerability profile.

### Field surveys

A series of surveys were conducted in 17 study areas in five countries in sub-Saharan African during 2013; Burkina Faso, Cameroon, Ethiopia, Uganda and Zambia. A previous review of tsetse density and trypanosome prevalence studies was the basis for the geographic focus of the study, identifying the selected countries as moderate to high risk AAT areas. The countries were also selected to cover a range of eco-regions and AAT epidemiology, in addition the ease of conducting fieldwork in the selected countries was taken into account. Within the countries, study areas were classified in terms of environment, including ecoregion and available information on AAT risk. A brief description of the study areas is given below and in Table 1.

**Table 1:** Brief description of the study areas based on previous tsetse & trypanosome information. Note: survey estimates in cattle were not necessarily from representative samples (hh’s = households)

Countries	Study areas	HH’s	Trypanosome cattle (%)	Trypanosome species	Tsetse species	Ref
Burkina Faso	Ioba & Sissili	123	4.3 % to 10 %	*T. vivax*, *T. congolense* and *T.brucei brucei*	*G. pallidipes gambiensis*, *G.tachinoides & G. morsitans submorsitans*	[4]
	Kénédougou	61				
	Léraba	41				
Cameroon	North Faro & Faro et Deo	131	35.1 %	*T. congolense*, *T. brucei & T. vivax*.	G*. m submortisans*, *G. fuscipes fuscipes & G. tachinoides*	[49, 50]
	South Faro	91	4.3 %			
	Mayo Rey	77	9.86 %			
Ethiopia	Goma & Setema	45	8.6 %–20.4 %	*T. congolense, T. vivax*, *T. b. brucei, T. evansi*	*G. fuscipes, G. pallidipes, G. m. submortisans, G. tachinoides, & G. longipennis*	[31, 51, 52]
	Goro & Cheha	36				
	Limmu Seka (East)	34				
	Limmu Seka (West)	36				
Uganda	Tororo	139	15.3 %	*T. vivax*, *T. congolense & T. brucei. rhodesiense*	*G. f. fuscipes*, *G. pallidipes & G. morsitans*	[21, 22]
	Buyende & Pallisa	78	27.5 %–35.7 %			
	Kumi & Ngora	83	29.0 %			
	Busia & Iganga	74				
Zambia	Lundazi – plateau	99		*T. vivax*, *T. congolense & T. brucei*	G*. m submortisans, G. pallidipes, G. breval papis, G. f. fuscipes & G. tachinoides*	[53]
	Lundazi – valley	57	17.8 %			
	Mambwe	54	28.4 %			

#### Burkina Faso

The main income in these study areas comes from rain fed agriculture with cattle utilised for draft power. Livestock rearing in extensive systems is also common; however trypanosomiasis is a constraint to livestock production in the area. AAT is endemic, and cattle owners report it as the most important disease in tsetse challenged areas [14]. Resistance to trypanocides is thought to be widespread, particularly isometamidium resistant *T. congolense*, and the first reports of trypanocide resistance came from these study areas [5]. The Léraba study area is crossed by 32,000 cattle per year from the North of Burkina Faso and Mali en route to markets in Côte d’Ivoire. In addition, during the dry season there is transhumance of Fulani cattle into the areas due to the availability of water points. Cattle entering the area may be highly susceptible to AAT.

#### Cameroon

The study was conducted in the Adamawa plateau which is the most important cattle rearing region in Cameroon. Here, white and red Fulani cattle are reared extensively, with a system of communal herding and Gudali (Sahelian Zebu) cattle are also important in the region. There is risk of AAT infection in at least two-thirds of the territory where 90 % of the cattle are found and the disease is one of the biggest limitations to the development of the cattle sector in Cameroon [15]. In 1995, at the end of the tsetse eradication campaign initiated by the government-founded ‘Mission spéciale pour l’éradication des glossines’(MSEG), the Faro et Déo division of the Adamawa plateau was divided into three zones: tsetse infested, tsetse cleared and a buffer zone between the two zones where all the cattle are treated with pyrethroids at regular intervals [16]. In 2010, a report from the Cameroonian government estimated that in tsetse infested zones, milk and meat sales were reduced by 50 % [15].

#### Ethiopia

Tsetse infest around 220,000 km^2^ of fertile land in south and southwestern parts of Ethiopia [8, 17]. AAT is thought to be the most important livestock disease in terms of economic development and influence on settlements [18]. AAT has also been reported as an important disease in other species especially in equines and goats [19]. The surveys were conducted in the Jimma zone of the Oromia region which is known for its large cattle numbers and the economy is also heavily reliant on crop production [18]. In this region cattle farmers attribute reductions in draft power and meat and milk offtake, increased calving intervals and mortalities and impacts on breeds kept and cattle management to AAT [18] .

#### Uganda

In Uganda the ‘tsetse belt’ runs from the highlands in southwestern Uganda across Lake Kyoga to north-eastern Uganda and at least 70 % of the entire country is thought to be infested with tsetse flies [20]. *T. vivax* is the most prevalent species of trypanosome in Ugandan cattle and *T. congolense* and *T. brucei rhodesiense* infections also occur [21, 22]. Following increases in human density, changes in land use, and a reduction in the wildlife population, Ugandan cattle are now considered the primary host of *T. b. rhodesiense* [23]. *T. b. rhodesiense* causes human African trypanosomiasis (HAT) or ‘sleeping sickness’ which is fatal if left untreated. The distribution of *T. b. rhodesiense* in Uganda has increased dramatically in the past 10 years; this is attributed to the restocking of infected cattle into naïve areas following military conflict in the late 1990’s [22]. Over 50 % of reported *T. b. rhodesiense* cases in the whole of Africa between 2000 and 2009 were from Uganda [24]. The study was conducted in the Southeast region of Uganda.

#### Zambia

The Luangwa valley runs through the Eastern Province of Zambia, with 3.84 million hectares of national park (46.9 %) and 0.41 million ha dedicated game management area the valley is an ecological niche for trypanosomes allowing vector-host interaction due to favourable conditions for tsetse in terms of vegetation, climate and abundance of wildlife hosts [25]. The study was conducted in Lundazi and Mambwe districts in the Eastern Province as there were reports of AAT, and cooperation with district veterinarians. Lundazi has a human population density of 22.4 people/km^2^ whilst Mambwe has a population density of approximately 13.4 people/km^2^. An increase in pressure on natural resources in the plateau area of the district has led to the relocation and expansion of the human population into the edges of the Luangwa valley expanding the wildlife-livestock interface. HAT cases have also occurred in the valley [26].

#### Selection of communities and households

Study areas were selected using random sampling from a sample frame of all communities within selected administrative divisions of each of the study countries. Within 195 communities 1,259 households across the five countries were visited during the course of the study. A household was defined as a group of people who usually reside and eat together. The head of the household was interviewed using a pre-piloted questionnaire which contained general questions about their herd, access to veterinary products and services, and livestock diseases. They were then asked questions specifically about AAT in an attempt to assess the relative importance of the disease to farmers. These questions focused on what is currently known, done and perceived with regards to AAT and its control. The study-unit for this analysis was the community; therefore the results from individual households were aggregated at community level.

### Selection of variables

The vulnerability of a community has been defined as a product of exposure, sensitivity and capacity to adapt when an extreme event takes place [27]. We consider exposure to AAT as the risk of AAT occurrence in the community which is influenced by climatic factors and the eco-epidemiology of the disease in the area. Sensitivity is defined by factors influencing the potential impact of AAT in the community, for example the susceptibility of cattle breeds, and the relative importance of cattle. Adaption refers to current measures to reduce the impact of the disease, either through the actions of farmers, governments or local authorities.

The first step for the MCA was to identify variables likely to be associated with the exposure, sensitivity and adaption to AAT in an area. This was done using existing literature and the available field data. It was based on two principal criteria: firstly, the relevance of variables to the objective of the assessment and secondly the completeness of data collected. The resulting variable selection is detailed in Table 2.

**Table 2:** Variables selected for inclusion in the MCA, and there classifications

	Variable	Classification
Exposure	Perceived incidence in the community	Whether the majority of cattle-owners reported AAT challenge as “rare”, “frequent” or “constant”
	Seasonality	Whether the majority of cattle-owners reported a seasonal effect of AAT
Sensitivity	Cattle breeds	Whether any cattle-owners owned *Bos Indicus* (trypanotolerant) or crossbreeds (partial trypanotolerance)
	Main cattle rearing systems	Whether farmers in the community were practicing tethering in addition to communal-grazing
	Tsetse control present	Whether at least 40 % of cattle-owning households reported existence of any form of tsetse control method in the community
	Herd size	Average herd size in the community classified into 3 bins of equal size
	Treatment failure	Whether the majority of farmers reported that treatment failure is “never” “rare” or “frequent”
	Treatment	Whether farmers report that they treat the disease themselves, or whether they rely on trained animal health workers, veterinary assistants or similar.
Capacity to adapt	Insecticide treated cattle (ITC)	Whether cattle-owning households reported the use of ITC as a measure of tsetse control in the community
	Tsetse control present	Whether cattle-owning households reported existence of any tsetse trapping in the community
	Farmer knowledge of AAT control	Whether the majority of farmers could recognise a picture of a tsetse trap and/or name tsetse control measures
	Losses to draft	Whether the majority of cattle-owning households report that AAT impacts their livelihood due to reductions in draft power
	Reported mortalities	Average number of mortalities reported by cattle owning households categorised into three bins
	Importance of cattle in the community	Whether the majority of cattle-owning households (>60 %) reported livestock as the primary agricultural income source, or if cropping or mixed farming systems are considered more important

### Multiple correspondence analysis (MCA) and cluster analysis

MCA is a data reduction technique (similar to factor analysis or principle components analysis) which allows complex patterns in a dataset of categorical variables to be identified. Briefly, MCA provides a graphic representation describing the relationships between categories of variables and creates factors which describe the variation in the data. This technique also allows variables exhibiting little variation between the communities to be excluded and those which vary the most between communities to be identified. This technique has previously been used to identify biosecurity profiles of farms [28, 29].

MCA was performed on the selected variables at community level using the Indicator method. The coordinates of each community were calculated on three dimensions explaining 47.1 % of the variance and HCA was then performed on the selected dimensions using Ward’s method to aggregate areas into relatively homogeneous subgroups or profiles. These profiles maximise inter-cluster variation and intra-cluster correlation. The analysis was performed using the package *FactoMineR* in R v. 3.0.1.

## Results and discussion

### Results of the multiple correspondence analysis

**Fig. 1:**
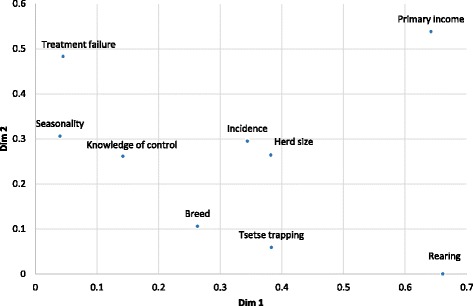
contribution of the variables to the formation of dimensions 1 and 2

**Fig. 2:**
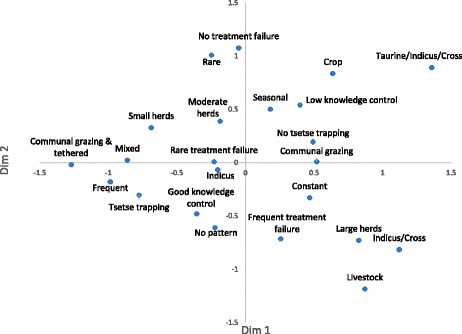
coordinates of each variable category on dimensions 1 and 2

Dimensions 1 and 2 from the results of the MCA of variables associated with AAT vulnerability are presented in Figs. 1 and 2. The importance of cattle in the community had the highest loadings on both dimensions with rearing method, herd size, perceived incidence and whether or not tsetse trapping was reported contributing the most to the formation of dimension 1. Frequency of treatment failure, whether cattle-owners felt the disease exhibited seasonal pattern, perceived incidence and herd size had the highest loadings on dimension 2. Mortality, who treats AAT (farmers’ vs. animal health workers) and whether ITC is used in the community were excluded because they had low loadings on all synthetic dimensions. The coordinates of the variables for dimension 1 and 2, which explained the largest percentage of the variation, are presented in Fig. 2.

### Results of the cluster analysis

**Fig. 3:**
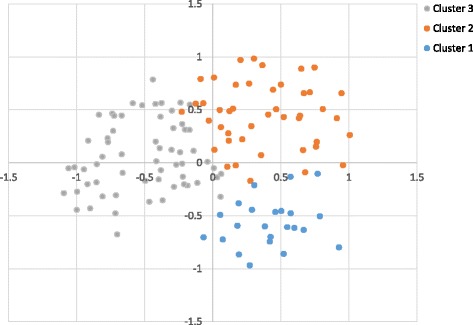
coordinates of each community on dimensions 1 and 2 and their cluster

The rural communities in this study were classified into three profiles or clusters (Fig. 3 and Table 3), which maximise inter vs. intra-cluster variation. Cluster 1 is characterised by the variables in the bottom right quadrant of Fig. 2, these communities reported constant AAT challenge (87.0 %) with no seasonal pattern (71.7 %). Most herds were trypanosensitive (87.0 %) (Red and White Fulani and some Gudali) and all practiced communal-grazing, although some communities reported keeping Zebu x Taurine cross-breeds in addition to local Zebu’s which have some trypanotolerance (13.0 %). These communities had larger herd sizes (average = 57) and cattle-owners were also more likely to report livestock farming as their primary source of income (87.0 %). Although the majority of communities appeared to have good knowledge of tsetse control (63.0 %), tsetse trapping was not reported in the area (80.4 %). These communities were most likely to report frequent treatment failure (69.6 %), and contained 91.1 % of the communities from Cameroon (Table 4).

**Table 3:** distribution of the variables retained in the final MCA and HCA, according to cluster

	Cluster 1	Cluster 2	Cluster 3
AAT challenge			
AAT constant	87.0 %	60.0 %	37.9 %
AAT frequent	8.7 %	6.7 %	37.9 %
AAT rare	4.3 %	33.3 %	24.1 %
Seasonality			
No pattern	71.7 %	16.7 %	50.6 %
Seasonal	28.3 %	83.3 %	49.4 %
Breeds kept			
Indicus	87.0 %	66.7 %	100 %
Indicus/Cross	13.0 %	1.7 %	-
Taurine/Indicus/Cross	-	31.7 %	-
Livestock rearing (day)			
Communal grazing	100 %	98.3 %	36.8 %
Communal & tethered	-	1.7 %	63.2 %
Average herd size			
Small (<7)	2.2 %	18.3 %	52.9 %
Moderate (7 to 13)	4.3 %	55.0 %	41.4 %
Large (>13)	93.5 %	26.7 %	5.7 %
Treatment failure			
No treatment failure	2.2 %	45.0 %	23.0 %
Rare treatment failure	28.3 %	31.7 %	46.0 %
Frequent treatment failure	69.6 %	23.3 %	31.0 %
Primary income			
Crop	2.2 %	83.3 %	10.3 %
Livestock	87.0 %	1.7 %	3.4 %
Mixed	10.9 %	15.0 %	86.2 %
Knowledge of tsetse control			
Good knowledge control	63.0 %	20.0 %	70.1 %
Low knowledge control	37.0 %	80.0 %	29.9 %
Tsetse trapping community			
No tsetse trapping	80.4 %	90.0 %	31.0 %
Tsetse trapping	19.6 %	10.0 %	69.0 %

**Table 4:** distribution of the communities according to country and cluster

Country	Cluster 1	Cluster 2	Cluster 3
Burkina Faso	4.8 %	95.2 %	-
Cameroon	91.1 %	8.9 %	-
Ethiopia	8.7 %	30.4 %	60.9 %
Uganda	2.7 %	6.6 %	90.4 %
Zambia	-	77.4 %	22.6 %

All of the communities which kept some trypanotolerant breeds (Boulé) and Métis crossbreeds were found in cluster 2 (31.7 %) and these were mainly communities located in Burkina Faso. This cluster is found in the top right quadrant of Figs. 2 and 3. Most communities practiced communal-grazing (98.3 %) and crop production tended to be the most important income source (83.3 %), followed by mixed farming. These communities had the lowest awareness of tsetse control measures with 80.0 % not identifying tsetse traps or naming tsetse control methods. Communities reported “never” experiencing treatment failure (45.0 %), although this is subjective. All communities in cluster 3 kept trypanosensitive Zebu breed but most of them practiced communal-grazing and tethering (63.2 %). The majority of farming was mixed i.e., livestock and crop farming of similar importance (86.2 %). Communities were most likely to report some tsetse control (69.0 %) and knowledge of AAT control tended to be good (70.1 %). Treatment failure was mostly reported as “rare” (46.0 %) in these communities. This cluster is described in the bottom left hand side of Figs. 2 and 3.

### Results of the cluster analysis: supplementary variables

We then investigated the difference between selected supplementary variables and membership of the different clusters (Table 5). The majority of communities did not report using ITC, (>80 % in every cluster). Communities in cluster 1 were most likely to believe that centralised governments (67.4 %) or NGO’s (17.4 %) were responsible for control. With cluster 2 and 3 more likely to name district officials or individuals.

**Table 5:** distribution of the supplementary variables according to cluster

	Cluster 1	Cluster 2	Cluster 3
ITC			
No ITC	82.6 %	85.0 %	83.9 %
ITC	17.4 %	15.0 %	16.1 %
Responsible for AAT control			
Communities	4.3 %	16.7 %	-
Individuals	6.5 %	33.3 %	24.1 %
District officials	4.3 %	28.3 %	46.0 %
Centralised governments	67.4 %	8.3 %	24.1 %
NGOs	17.4 %	13.3 %	5.7 %
Diagnosis			
Farmers	89.1 %	63.3 %	50.6 %
Trained	10.9 %	36.7 %	49.4 %
Treatment			
Farmers	73.9 %	43.3 %	37.9 %
Trained	26.1 %	56.7 %	62.1 %
Reasons for treatment failure			
Misdosing	21.7 %	11.7 %	16.1 %
Drug quality	60.9 %	20.0 %	37.9 %
Misdiagnosis	30.4 %	25.0 %	28.7 %
Resistance	30.4 %	63.3 %	57.5 %
Total cost AAT			
Low (<$15)	17.4 %	28.3 %	42.5 %
Medium ($15 to $55)	19.6 %	40.0 %	41.4 %
High (>$55)	63.0 %	31.7 %	16.1 %
Cattle mortalities			
Low	4.3 %	4.9 %	4.7 %
Medium	39.1 %	35.0 %	26.7 %
High	56.5 %	61.7 %	67.4 %
Draught losses			
No	67.4 %	53.3 %	49.4 %
Yes	32.6 %	46.7 %	50.6 %

When asked about diagnosis and treatment, farmers were most likely to be responsible for both in cluster 1 (diagnosis: 89.1 %, treatment: 73.9 %). With between a half and two-thirds of communities reportedly using trained individuals for diagnosis and treatment in clusters 2 and 3. Trypanocidal resistance was reported as a possible reasons for treatment failure in clusters 2 (63.3 %) and 3 (57.5 %). Drug quality was also a concern of farmers in clusters 1 (60.9 %) and 3 (37.9 %). Communities appeared to spend the most diagnosing and curatively treating AAT in cluster 1 with 63.0 % reportedly spending an average of 55 or more US$ in the past two years. Communities in cluster 2 appeared to have incurred the lowest costs with 42.5 % spending less than 15 US$ over the same time period.

**Fig. 4:**
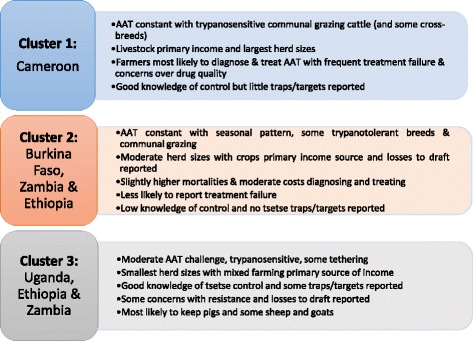
overall summary of the results of selected communities in five sub-Saharan African countries based on determinants of AAT vulnerability

In terms of herd sizes, cluster 1 communities tended to have larger numbers of cows (82.6 % > 10), castrated males (47.8 % > 2), uncastrated males (71.7 % > 4) and calves (80.4 % > 4). Sheep were kept by around 90 % of these communities but they were less likely to keep goats (43.5 % = zero) and pigs (91.3 % = 0). Cluster 2 was mostly composed of medium sized (4 to 10 adult female cows) herds. Most households in this cluster also kept sheep and goats. Most households in cluster 3 had small herds of up to three adult female cows (57.5 %) with the rest keeping 4 to 10, around half had sheep and were more likely to have goats than households compared to the other two clusters. These communities were the most likely to keep pigs (67.8 %), with 53.3 % of farmers in cluster 2 also keeping pigs. A summary of the results is given in Fig. 4.

The results from this study were then compared to previous surveys available in the study areas (Table 1). Cluster 1 appeared to be vulnerable to AAT and prevalence’s as high as 35.4 % have been reported in the Adamawa Plateau study areas [30]. Burkina Faso study areas appeared to have the lowest AAT prevalence, and these communities were the most likely to be using trypanotolerant breeds [4]. Some communities in Uganda (Cluster 3) reported rare AAT occurrence, although others reported it as frequent or constant. Previous prevalence estimates of AAT in Uganda were higher than the majority of other study areas [22], however, these studies were conducted in markets and may represent a higher risk population. Cluster 3 also included Ethiopian communities, in some areas close to the study region farmers have ranked the importance of the disease as “moderate” and prevalence estimates were around 8.7 %. This is likely due to the establishment of the Southern Tsetse Eradication Programme (STEP) [31]. Further work is needed to assess how farmers’ perceptions of the disease compare with the epidemiology of the disease in different areas.

Historically, large-scale efforts to control trypanosomiasis have focussed on elimination of the vector which requires considerable investment and detailed planning. In addition, sustained elimination is only possible when the total tsetse population is addressed, which requires either an isolated population, or the sequential eradication of a full tsetse belt [32, 33]. Coordinated efforts between communities within and between countries are needed as well as strong political and financial support [13]. As a result, many previous elimination efforts have not been successful, or AAT has re-emerged after funding for control has waned [32]. In both Burkina Faso and Cameroon study areas, successful suppression of tsetse was achieved in the past through integrated control programmes managed by specialist government institutions, however, in both cases reinvasion occurred as control efforts were not sustained [16, 34].

In areas where sustained suppression is not currently feasible, understanding of how cattle-owners are affected by AAT and their efforts to manage the disease is critical to the design of suitable locally-adapted control programmes [13]. The variables considered in the analysis were assumed to describe the vulnerability of a community to AAT and this information could help target communities to receive support to implement control options. Given the heterogeneity across communities studied, in some communities farmers may be successful at managing the disease on their own. In clusters 2 and 3 farmers have developed some strategies to manage the disease, such as restricted grazing or the use of trypanotolerant cattle. Whereas for some cattle-owning communities, for example those in cluster 1 experiencing treatment failure or high mortalities, the vulnerability may be so high that considerable external support and investment is needed to reduce the trypanosome burden. However, the study used data provided by cattle-owners and did not investigate parasitological prevalence or surveys of tsetse. The results should be integrated with other data e.g., recent tsetse and unbiased cattle prevalence surveys and interviews with managers of AAT control to investigate the relationship between these data sources and the burden reported by cattle-owners.

Farmers are heavily reliant on chemotherapy for AAT control [28, 35]. Trypanocide use was ubiquitous throughout the study areas and therefore showed no association with any particular cluster. In these communities there appeared to be a lack of vector control. Alternative control measures such as ITC or live bait technologies, screens, traps and targets may represent a cost-effective alternative to trypanocides [18]. Although in some communities there may have been a lack of awareness of these tsetse control methods (particularly in cluster 2), farmers’ also face a collective-action dilemma when it comes to the financing and organising of community-level interventions. These areas may benefit from governmental or institutional interventions to provide community-level tsetse control or to help mobilise communities to organise themselves and adopt technologies from which all community members may benefit. Cost-recovery schemes have had some success, but depend on financial resources of the farmers and the perceived benefits of the initiative [36]. In Ethiopia a cost-recovery scheme was initiated in an area with high trypanocide resistance consisting of monthly pour-on application with cypermethrin and chemotherapy [37]. In the study area an average decrease of 57 % in calf mortality (including still births) by 12 months of age and an increase of 8 % in the body weight of adult males was observed [37], suggesting that the scheme was successful.

In some communities cattle production is unlikely to be sustainable due to the high tsetse-trypanosome burden and lack of herd or community-level control of the disease due to lack of resources or technical capacity [13]. In these communities, farmers explore alternative sources of income. This is the case in the valley study area of Lundazi (cluster 2), where access to markets and veterinary services are poor and farmers co-exist with an expanding wildlife and tsetse population. There are few-cattle owning households in this region, and primary source of household income tends to be from crop production. Here communities tend to keep trypanosensitive Angoni breeds whose draft power provision is greatly reduced by AAT. Some farmers in Burkina Faso managed the disease by using the trypanotolerant Métis or Baoule cattle breeds; similarly, the introduction of trypanotolerant breeds may be of benefit in Zambia. However, trypanotolerant breeds are considered to have reduced traction which is the main use of cattle in this area, although this may be offset in areas with high morbidity and mortalities in trypanosensitive draft animals [38].

Where seasonality allows, an alternative management strategy to reduce the risk of AAT is to only graze in tsetse infested areas in certain times of the year [35]. In the case of Cameroon, which was mainly represented by cluster 1, farmers manage the disease by only entering the valley region during the dry season where AAT risk is at its lowest [16]. The majority of farmers kept trypanosensitive Fulani and Gudali cattle, therefore perhaps the use of trypanotolerant cattle would also reduce the impact here. Although farmers in the region have a strong cultural preference for the traditional Fulani breeds. Trypanotolerant breeds such as N’Dama of West Africa may have comparable productivity in terms of meat and milk to trypanosensitive breeds in areas where AAT burden is high, and the majority of cattle in the Cameroonian study areas are kept for this purpose [39]. Few communities kept a large proportion of trypanotolerant breeds, and those that did were mainly communities of Burkina Faso in cluster 2, it is estimated that there were 11.68 million trypanotolerant cattle in 1998 of which 11 million were in West Africa and 0.68 million in Central Africa [40].

Communities in cluster 1 reported no tsetse control, despite the Mission spéciale pour l’éradication des glossines (MSEG) running low level control operations for many years [16]. However, there are only a small number of traps and targets in the cleared area and buffer zone in the Faro et Deo region of Cameroon (personal communication: MSEG). The MSEG also report some bi-annual trypanocide and ITC campaigns before and after transhumance [16]. However, the majority of cattle-owners are responsible for AAT diagnosis and administration of trypanocidal drugs. The farmers in these communities reported frequent treatment failures. Around a third of communities here, and in cluster 3, attributed treatment failure to misdiagnosis. The evidence as to whether farmer-based diagnosis and treatment is satisfactory is conflicting and will vary between communities depending on experiences and training received [3, 35, 41, 42]. In a previous study in Busia in Kenya farmers underestimated the bodyweight of 85.7 % of cattle by an average of 46.9 %, which has serious implications for the development of trypanocide resistance [43].

Ideally, livestock owners should be encouraged to use trained veterinarians and veterinary assistants to diagnose and treat the disease. However, an estimated 35 million trypanocide doses are administered every year, large numbers of animals can be affected in certain seasons and many communities with the disease are in more remote areas close to national parks or game reserves where access to veterinary services may be reduced [1]. Training of farmers and selected individuals in the community (CAHWs) can be highly effective to improve diagnosis and ensure correct dosing, although this can be expensive [35]. Following privatisation of the veterinary services in many SSA countries, CAHWs are increasingly used by livestock owners, particularly in remote communities [44]. Providing tools such as weigh-bands to estimate correct dosing for cattle could also be of use in these communities [43].

Trypanocide resistance was the main reason attributed to treatment failure in cluster 2, and second most cited reason in clusters 1 and 3. Trypanocide resistance has been reported in most of the regions studied within the five countries [5, 18, 45]. Trypanocide resistance may be linked to under-dosing, or drugs containing insufficient quantities of the active compound. Problems with the drugs were mentioned as a major reason of treatment failure by cluster 1 communities. A study found that 69 % of trypanocides purchased form legal and illegal markets in Cameroon failed to comply with pharmaceutical requirements, with 42 % due to insufficient quantities of the active ingredient [46].

This study only considered AAT and not HAT, in areas where both diseases overlap efforts between animal and public health officers should be coordinated. For example, the Stamp Out Sleeping Sickness (SOS) campaign was a public-private partnership designed to target the cattle reservoir of *T. b. rhodesiense* in newly affected areas of Northern Uganda by block treating >180,000 head of cattle [23]. Communities in Uganda were also the most likely to keep pigs and the prevalence of *T. brucei* of pigs in the Iganga study area has been reported to be around 8.1 % [47]. Pigs were also important in cluster 2 and have been shown to be affected by *T. congolense* in the Zambian study area, in addition there are an abundance of wildlife hosts for AAT here [25, 48]. Highlighting the need to consider other livestock and wildlife species present when designing AAT control programmes [47].

Vulnerability to AAT is dynamic and complex and contains some level of judgement. The current study is solely based on the experience of farmers, who are usually not involved in the design of control programmes but who suffer the impacts of the disease. One identified key failure from reviews of past control programmes was the inability to transfer responsibility for the control of AAT to cattle-owners once the programme has ceased [36]. Further work is needed to elaborate on reasons why this may be the case. In light of this work a review of AAT control programmes in the countries is now being conducted investigating their success and sustainability [36]. This work also includes interviews with managers of AAT control programmes. In this study engaging with farmers helped raise awareness about the disease and transferred knowledge regarding its control. Soliciting farmers’ views and experiences may motivate them to take action themselves and cooperate with externally-led control programmes, increasing their effectiveness. For example, in one of the study area in Zambia, few participants were able to identify a picture of tsetse trap (8.9 %). Following the interviews some farmers said they had seen these traps, but had not known their purpose and that they are often destroyed for their material.

## Conclusions

This study identified three clusters of community vulnerability profiles within the five countries studied, based on farmers’ beliefs with respect to trypanosomiasis control. Further work relating this information to entomological, disease prevalence and data from interviews with AAT control managers is needed to triangulate these results. However, these findings already provide an indication of the ways in which community level variables may impact upon the need, suitability and effectiveness of disease control programmes. Detailed descriptive reports at household-level are also being published, which include information on the demand and willingness to pay for novel treatments and diagnostics under development. Results of this work will be used to develop tailored recommendations, to reduce the impact of AAT in these communities. Including the integration of novel treatments and diagnostics with new and existing programmes, should they become available. The results may also be applicable to other communities with similar profiles to those identified here.

## Abbreviations

AAT: Animal African trypanosomiasis
HAT: Human African trypanosomiasis
MCA: Multiple correspondence analysis
HCA: Hierarchical cluster analysis
MSEG: Mission spéciale pour l’éradication des glossines
CAHW: Community animal health workers
